# Cost-effectiveness analysis of irinotecan hydrochloride liposome in combination with 5-fluorouracil and leucovorin in locally advanced or metastatic pancreatic ductal adenocarcinoma

**DOI:** 10.3389/fphar.2025.1596658

**Published:** 2025-11-12

**Authors:** ShaoQing Zhan, PanFeng Feng

**Affiliations:** 1 Department of Pharmacy, Affiliated Hospital 2 of Nantong University, and First People’s Hospital of Nantong City, Nantong, Jiangsu, China; 2 Nantong Key Laboratory of Innovative Research on Rheumatology and Immunology, Nantong, Jiangsu, China; 3 Nantong Clinical Medical College of Kangda College of Nanjing Medical University, Nantong, Jiangsu, China

**Keywords:** cost-effectiveness analysis, irinotecan hydrochloride liposome, second-line treatment, locally advanced or metastatic pancreatic ductal adenocarcinoma, partitioned survival model

## Abstract

**Objective:**

This study aims to evaluate the cost-effectiveness of irinotecan hydrochloride liposome in combination with 5-fluorouracil and leucovorin (5-FU/LV) as a second-line treatment in locally advanced or metastatic pancreatic ductal adenocarcinoma, from the perspective of the healthcare system in China.

**Methods:**

A partitioned survival model was developed based on data from PAN-HEROIC-1 clinical trial (NCT05074589) and relevant literature. The simulation horizon was set at 5 years, with a cycle length of 2 weeks. Costs and utility values were discounted at an annual rate of 5%. Quality-adjusted life years (QALYs) served as the primary outcome measure, and the incremental cost-effectiveness ratio was calculated to compare irinotecan hydrochloride liposome plus chemotherapy regimen (experimental group) with the 5-FU/LV regimen (control group). One-way sensitivity analysis and probabilistic sensitivity analysis were performed to assess the robustness of the results.

**Results:**

The results revealed that the ICER for the experimental group compared to the control group was ¥1,271,796.38/QALY, exceeding the willingness-to-pay (WTP) threshold of three times China’s per capita gross domestic product (GDP) in 2023. One-way sensitivity analysis indicated that parameters such as utility value during progression-free survival, body surface area, the cost of irinotecan hydrochloride liposome and the utility value during disease progression significantly influenced the model outcomes. Probabilistic sensitivity analysis demonstrated that the probability of the irinotecan hydrochloride liposome being cost-effective was 0.

**Conclusion:**

When using three times per capita GDP of China in 2023 as the WTP threshold, the irinotecan hydrochloride liposome plus chemotherapy regimen is not considered cost-effective compared to the standard 5-FU/LV regimen.

## 1 Introduction

Pancreatic cancer is a prevalent and highly aggressive malignancy of the digestive system, characterized by its insidious onset and high degree of malignancy. The majority of patients are diagnosed at an advanced stage, leading to a high rate of recurrence, metastasis, and mortality. The 5-year survival rate for pancreatic cancer patients is less than 5% (Bengtsson et al., 2020), significantly lower compared to other types of malignancies. In 2020, pancreatic cancer accounted for 466,000 deaths globally, ranking it seventh among all cancer-related deaths (NIH, 2025). Currently, chemotherapy remains the primary treatment modality for locally advanced or metastatic pancreatic cancer; however, patients face significant challenges, including short overall survival and poor quality of life. For locally advanced or metastatic pancreatic ductal adenocarcinoma (PDAC), commonly employed chemotherapy regimens include the AG regimen (albumin-bound paclitaxel combined with gemcitabine) and the FOLFIRINOX regimen (a combination of leucovorin, 5-fluorouracil, irinotecan, and oxaliplatin) (Burris et al., 2023; Conroy et al., 2011; Von Hoff et al., 2013; St et al., 1999; Cui et al., 2024a; Cui et al., 2024b). While these treatments demonstrate initial efficacy, most patients ultimately experience disease progression or recurrence, underscoring the critical need for effective second-line treatment strategies following first-line therapy.

Irinotecan, an effective inhibitor of topoisomerase I, plays a crucial role in numerous classical chemotherapy regimens. However, the traditional formulation of irinotecan is associated with reduced efficacy and increased side effects due to its pharmacological properties. A randomized, double-blind, phase III clinical trial, PAN-HEROIC-1 (NCT05074589), conducted among the Chinese population, assessed the efficacy and safety of irinotecan hydrochloride liposome in combination with fluorouracil/leucovorin (5-FU/LV) versus placebo plus 5-FU/LV in patients with unresectable, locally advanced, or metastatic pancreatic ductal adenocarcinoma (PDAC) who had previously received gemcitabine-based therapy (Liu, 2020). The study results demonstrated that compared with the standard chemotherapy regimen, the irinotecan hydrochloride liposome combined chemotherapy regimen significantly extended median overall survival (mOS: 7.4 months vs. 5.0 months) and median progression-free survival (mPFS: 4.2 months vs. 1.5 months). The irinotecan hydrochloride liposome combined chemotherapy has been recommended as a second-line treatment option for PDAC in the “Pancreatic Cancer Diagnosis and Treatment Guidelines (2022 Edition)”. However, the economic efficiency of this regimen within the Chinese healthcare context remains unclear. Therefore, this study evaluates the cost-utility of the irinotecan hydrochloride liposome combined chemotherapy regimen as a second-line treatment for PDAC from the perspective of China’s health system based on the PAN-HEROIC-1 trial, providing a reference for the rational selection of clinical medication regimens.

## 2 Materials and methods

### 2.1 Target population and treatment regimen

The target population and treatment regimen of this study are consistent with those of the PAN-HEROIC-1 clinical trial. Specifically, this study includes patients aged 18 years or older with histologically confirmed advanced or metastatic pancreatic ductal adenocarcinoma (PDAC) who have either failed or are intolerant to first-line gemcitabine-based chemotherapy. Patients with central nervous system metastases or other malignancies within the past 5 years were excluded. A total of 298 patients with advanced PDAC were randomly assigned in a 1:1 ratio to the irinotecan hydrochloride liposome plus chemotherapy regimen (experimental group) and the placebo plus chemotherapy group (control group). Patients in both groups received either irinotecan hydrochloride liposome (60 mg/m^2^ of irinotecan hydrochloride anhydrous, equivalent to 56.5 mg/m^2^ of free base) or placebo, combined with 5-fluorouracil (5-FU, 2000 mg/m^2^) and leucovorin (LV, 200 mg/m^2^), administered intravenously every 2 weeks. Treatment continued until disease progression (PD), the occurrence of unacceptable toxicity, or withdrawal from the study for other reasons. Upon disease progression, patients discontinued the current treatment regimen and transitioned to subsequent-line therapy. Given that the PAN-HEROIC-1 trial did not provide detailed information on specific post-progression treatments, it is assumed that all patients received best supportive care. It was observed that a higher proportion of patients in the placebo group received post-progression antitumor therapy compared to the intervention group (68.5% vs. 51.7%) (Cui et al., 2024b). Consequently, applying a uniform Best Supportive Care (BSC) cost assumption to both groups in our model may lead to an underestimation of the actual costs in the placebo group, thereby potentially resulting in an overestimation of the Incremental Cost-Effectiveness Ratio (ICER). Recognition of this potential bias represents a key limitation of this study, the implications of which will be further analyzed in the Discussion section.

Our analysis was grounded in existing literature and did not entail any new studies involving human participants or the use of human or animal tissues/samples by the authors. This study strictly followed the Consolidated Health Economic Evaluation Reporting Standards (CHEERS) for reporting health economic evaluations (Husereau et al., 2022). As this study is entirely based on previous research (Liu, 2020) and publicly available data, it does not include any new research involving human participants or animals by any of the authors, and therefore does not require approval from an independent ethics committee. The data is freely available by searching for the keyword NCT05074589 on https://clinicaltrials.gov/.

### 2.2 Model structure

**FIGURE 1:**
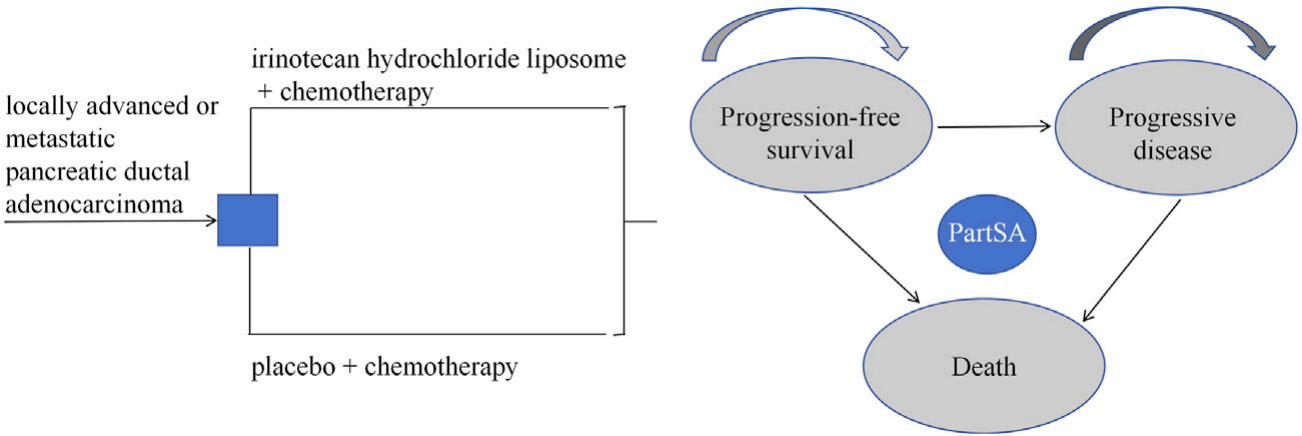
Partition survival model. PartSA, partitioned survival approach.

Based on the disease progression process, a partitioned survival model was constructed using Excel 2021 and R 4.4.1 software. This study defines three mutually exclusive health states: progression-free survival (PFS), progressive disease (PD), and death. The model structure is illustrated in Figure 1. The partitioned survival model extrapolates the Kaplan-Meier curves for progression-free survival (PFS) and overall survival (OS) to directly calculate the proportion of patients alive in each health state, without using transition probabilities between states. It is assumed that all patients begin in the PFS state, and each patient can only occupy one health state per cycle. Transitions between these three states are unidirectional and irreversible. The model cycle aligns with the treatment cycle, both set at 2 weeks. Given the poor prognosis of newly diagnosed advanced PDAC patients, with a 5-year survival rate of less than 5% post-diagnosis (Bengtsson et al., 2020), this study sets the simulation time horizon to 5 years. The primary outcome measures of the model include total cost, quality-adjusted life years (QALYs), and incremental cost-effectiveness ratio (ICER). In this study, the willingness-to-pay (WTP) threshold is set at three times China’s per capita GDP for 2023, equivalent to ¥268,074/QALY. According to the “Chinese Guidelines for Pharmacoeconomic Evaluation (2020)” (Liu, 2020), costs and health outcomes were discounted at a rate of 5%, and sensitivity analysis of the discount rate was conducted within the range of 0%–8%.

### 2.3 Survival analysis

When the simulation time is within the follow-up period, the distribution of individuals across health states is directly derived from the overall survival (OS) and progression-free survival (PFS) curves. The proportion of individuals in the PFS state is provided by the PFS curve, while the proportion in the death state is calculated as (1 - overall survival rate). The proportion in the progressive disease (PD) state is determined by the difference between the OS and PFS survival rates. When the simulation time exceeds the follow-up period, the survival function is estimated using a parametric approach. Specifically, this study utilized WebplotDigitizer 4.7 software to extract data points from the survival curves of the PAN-HEROIC-1 trial (Cui et al., 2024b). Subsequently, R 4.4.1 software was employed to reconstruct the PFS and OS curves. Survival analysis fitting was performed on the reconstructed individual-level data using Exponential, Weibull, Gompertz, Log-normal, and Log-logistic distributions. Finally, the optimal fitting distribution was selected based on the Akaike information criterion (AIC) and Bayesian information criterion (BIC), supplemented by visual Inspection.

**FIGURE 2:**
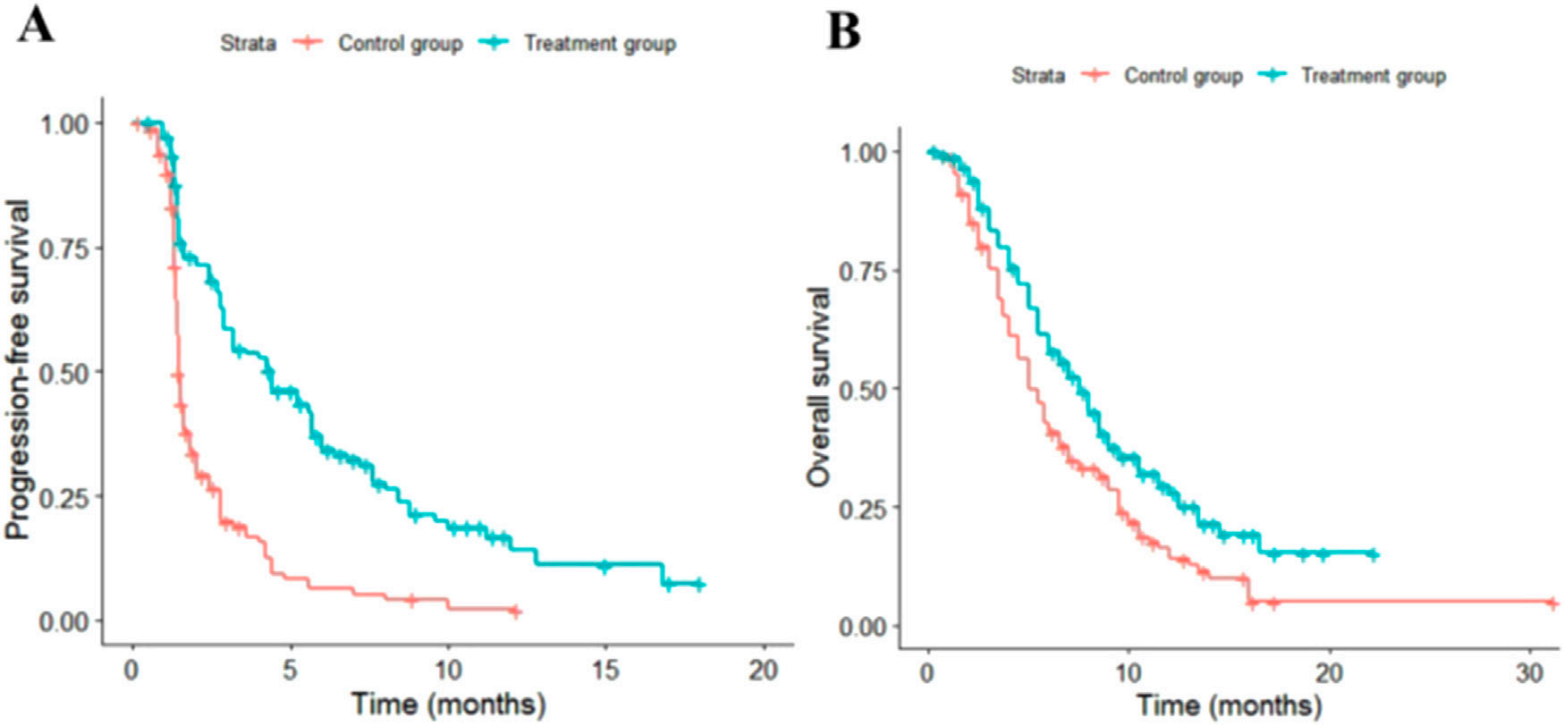
Fitted survival curves. **(A)** Fitted PFS curve. **(B)** Fitted OS curve.

The fitting results for different parametric distributions of the PFS and OS curves for the two patient groups are presented in Table 1. Based on these results, this study selected Log-logistic and lognormal distributions to fit the PFS and OS curves of the two treatment regimens, respectively. The distribution parameters of the survival curves were estimated using R 4.3.1 software, allowing for the calculation of the patients’ survival functions. The distribution parameters of the survival curves are summarized in Table 2, while the fitted survival curves are illustrated in Figure 2.

**TABLE 1:** AIC and BIC values for PFS curve and OS curve fitting in experimental group and control group.

Curve of treatment		Exponential	Gompertz	Weibull	Loglogistic	Lognormal
PFS curve (experimental group)	AIC	569.910	571.750	565.757	552.103	544.976
	BIC	572.914	577.758	571.765	558.111	550.984
PFS curve (control group)	AIC	456.434	457.787	434.467	366.539	376.909
	BIC	459.438	463.794	440.475	372.546	382.917
OS curve (experimental group)	AIC	665.642	660.018	646.443	637.571	637.226
	BIC	668.646	666.026	652.451	643.579	643.234
OS curve (control group)	AIC	725.830	724.250	708.308	694.312	692.377
	BIC	728.834	730.258	714.316	700.320	698.384

**TABLE 2:** Optimal fitting distribution and distribution parameters of Kaplan-Meier curve.

Kaplan-meier curve	Optimal fitting distribution	Mean	Standard deviation (SD)
PFS curve (experimental group)	Lognormal	μ = 1.443,565	σ = 0.930,274
PFS curve (control group)	Loglogistic	Ƴ = 3.09203	λ = 1.72203
OS curve (experimental group)	Lognormal	μ = 2.012807	σ = 0.832,437
OS curve (control group)	Lognormal	μ = 1.681,945	σ = 0.806,061

### 2.4 Cost and utility values

This study adopts the perspective of the Chinese healthcare system for evaluation. The system is characterized by a basic medical insurance scheme as its main component, with the National Healthcare Security Administration (NHSA) uniformly managing the payment standards for medicines and medical services. Only direct medical costs were considered, including treatment costs for different courses, laboratory test costs, radiological examination costs, and adverse reaction costs. Drug-related adverse reactions included in the cost analysis were limited to those graded ≥3 with an incidence rate ≥5%, specifically anemia, neutropenia, leukopenia, and elevated transaminases. It was assumed that all patients received best supportive care after progressive disease (PD). Drug costs were based on the average winning bid prices published by Yaozh (https://www.yaozh.com/) in 2023. The baseline body surface area (1.72 m^2^) used in the model for calculating drug costs was derived from a Chinese population study (Wu et al., 2012). Health utility parameters, adverse reaction costs, and costs for laboratory tests and radiological examinations were derived from previously published literature (Zhu et al., 2023; Ding et al., 2021; Qin et al., 2018; Attard et al., 2014). All cost data in this study were modeled using a Gamma distribution, while utility values and adverse reaction incidence rates followed a Beta distribution (Table 3).

**TABLE 3:** Model parameters and distribution types.

Variable	Baseline value	Minimum	Maximum	Distribution	Parameters for probability distributions	References
Costs (¥)						
Irinotecan hydrochloride liposome/mg	183.22	146.57	219.86	Gamma	α = 25.3, β = 7.24	https://www.yaozh.com
Calcium folinate/mg	0.2504	0.5098	0.7648	Gamma	α = 15.2, β = 0.0165	https://www.yaozh.com
Fluorouracil/mg	0.1176	0.0941	0.1411	Gamma	α = 30.1, β = 0.0039	https://www.yaozh.com
Radiological examination	1627.79	1302.23	1953.35	Gamma	α = 18.5, β = 88.0	11
Anemia	3536.60	2829.28	4243.92	Gamma	α = 12.7, β = 278.5	12
Decreased white blood cell count	3099.60	2479.68	3719.52	Gamma	α = 14.2, β = 218.3	12
Decreased neutrophil count	3069.67	2455.74	3683.60	Gamma	α = 13.8, β = 222.4	12
Elevated transaminase levels	415.76	332.60	498.91	Gamma	α = 10.5, β = 39.6	13
Optimal supportive care	1747.58	1398.07	2097.10	Gamma	α = 20.3, β = 86.1	11
Incidence of adverse reactions/%						
Elevated transaminase (study group)	19.1	15.28	22.92	Beta	α = 23, β = 97	8
Anemia (study group)	6.1	4.88	7.32	Beta	α = 9, β = 138	8
Decreased neutrophil count (study group)	12.9	10.32	15.48	Beta	α = 15, β = 101	8
Decreased white blood cell count (study group)	8.2	6.56	9.84	Beta	α = 11, β = 123	8
Elevated transaminase levels (control group)	11.4	9.12	13.68	Beta	α = 14, β = 109	8
PFS	0.85	0.68	1.00	Beta	α = 85, β = 15	14
PD	0.73	0.584	0.876	Beta	α = 73, β = 27	14
Discount rate/%	5	0	8	Beta	α = 5, β = 95	9
*Body surface area*	1.72	1.38	2.06	Gamma	α = 16, β = 0.1075	15

### 2.5 Sensitivity analysis

To assess the robustness of the model, this study performed both one-way and probabilistic sensitivity analyses. In the one-way sensitivity analysis, each parameter was varied by ±20% from its baseline value; the discount rate was set within a range of 0%–8%, in accordance with the “Chinese Guidelines for Pharmacoeconomic Evaluation (2020)” (Liu, 2020). The influence of each parameter on the incremental cost-effectiveness ratio (ICER) was evaluated based on these variations, and the results were visualized using a tornado diagram. For the probabilistic sensitivity analysis, it was assumed that cost parameters followed a Gamma distribution, while utility values, adverse event rates, and discount rates followed a Beta distribution. A total of 10,000 Monte Carlo simulations were conducted to sample from the distributions of each parameter, and the findings were presented as a cost-effectiveness acceptability curve.

## 3 Results

### 3.1 Base case results

The results of the base case analysis are presented in Table 4. As shown in Table 4, compared with the control group, the study group can provide an additional 0.21 QALYs for PDAC patients, but it also incurs a significantly higher treatment cost. The ICER of the study group relative to the control group is ¥1,271,796.38 per QALY, which far exceeds the willingness-to-pay (WTP) threshold of three times China’s 2023 per capita GDP (¥268,074 per QALY). Therefore, this intervention is not considered cost-effective.

**TABLE 4:** Base-case results of the model.

Group	Costs (¥)	QALYs	ΔCosts (¥)	ΔQALYs	ICER (¥/QALY)
Study group	278647.36	0.70	261300.56	0.21	1271796.38
Control group	17346.8	0.49			

ICER, incremental cost-effectiveness ratio; QALYs, quality-adjusted life-years.

### 3.2 Scenario analysis and threshold analysis

Scenario Analysis: A scenario analysis was conducted by extending the model simulation time horizon from 5 years to 10 years. The results showed that the total costs in the intervention and control groups increased to ¥280,419.77 and ¥17,950.80, respectively, while the total utilities increased to 0.71 QALYs and 0.50 QALYs, respectively. The incremental cost-effectiveness ratio (ICER) was ¥1,266,032.90 per QALY, which was very close to the base-case result and still far exceeded the WTP threshold.

Threshold Analysis: The threshold analysis indicated that at the WTP threshold of three times China’s per capita GDP (¥268,074 per QALY), the price of irinotecan hydrochloride liposome (HR070803) would need to be substantially reduced from the current ¥183.22 per mg to approximately ¥31.9 per mg (i.e., a price reduction of about 82.6%) for the combination regimen to be considered cost-effective. This finding provides a clear quantitative reference for future health insurance price negotiations.

### 3.3 Sensitivity analysis

**FIGURE 3:**
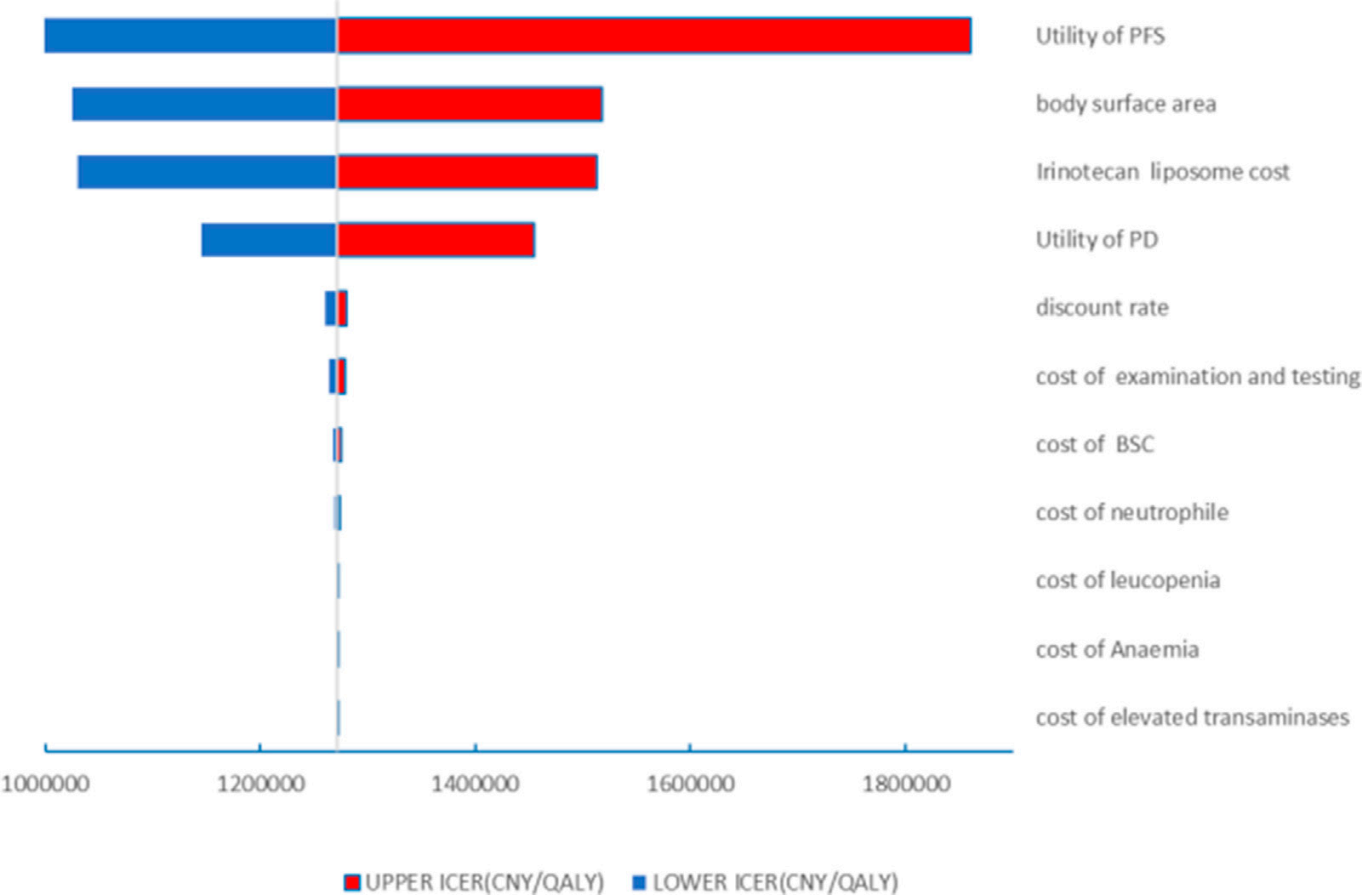
Tornado diagram for univariate sensitivity analyses.

**FIGURE 4:**
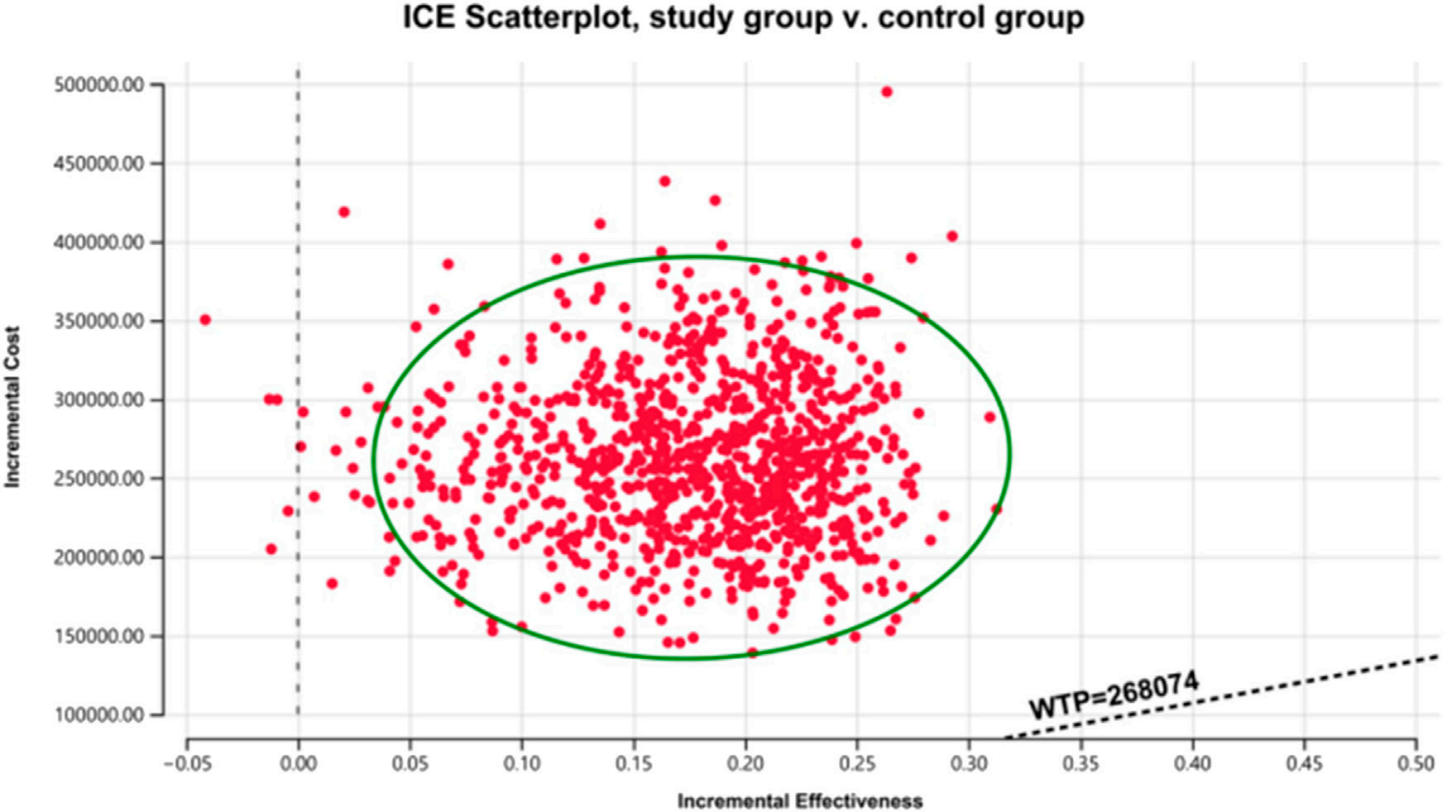
Cost-effectiveness scatter plot.

**FIGURE 5:**
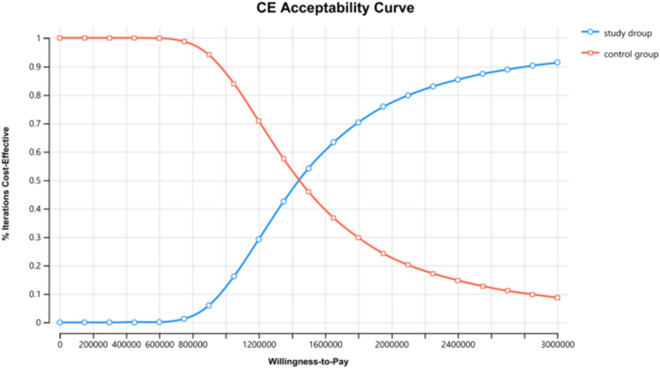
The cost-effectiveness acceptability curves for probabilistic sensitivity analyses.

The results of the one-way sensitivity analysis in the PartSA model indicated that the four parameters with the greatest impact on the ICER were the utility value of the PFS state, body surface area, the price of irinotecan hydrochloride liposome, and the utility value of the PD state (Figure 3). The results of the probabilistic sensitivity analysis further validated the robustness of the base-case analysis. As shown in Figure 4, the incremental cost-effectiveness scatterplots generated from 10,000 Monte Carlo simulations all fell above and to the right of the line representing the willingness-to-pay (WTP) threshold (¥268,074 per QALY). This indicates that in the vast majority of simulations, the incremental cost of the irinotecan hydrochloride liposome combination regimen far exceeded the value corresponding to the threshold. The cost-effectiveness acceptability curve (Figure 5) showed that the probability of the irinotecan hydrochloride liposome regimen being cost-effective was 0% at the WTP threshold of ¥268,074 per QALY. Even when the WTP threshold was raised to ¥1,200,000 per QALY (approximately 4.5 times the per capita GDP), the probability of it being cost-effective remained below 50%. Collectively, these results demonstrate that the conclusion of the base-case analysis—that the regimen is not cost-effective at its current price—is robust to uncertainty in the model parameters.

## 4 Discussion

This study, based on data from the PAN-HEROIC-1 phase III clinical trial, is the first to evaluate the cost-effectiveness of irinotecan hydrochloride liposome (HR070803) combined with 5-FU/LV as a second-line treatment for advanced pancreatic ductal adenocarcinoma (PDAC) from the perspective of the Chinese healthcare system. The base-case analysis indicated that compared to the 5-FU/LV control group, the HR070803 regimen provided a health benefit of 0.21 quality-adjusted life years (QALYs) but incurred additional medical costs of ¥261,300.56, resulting in an incremental cost-effectiveness ratio (ICER) of ¥1,271,796.38 per QALY. This figure substantially exceeds the willingness-to-pay (WTP) threshold of three times China’s per capita GDP (¥268,074 per QALY). Both one-way and probabilistic sensitivity analyses confirmed the robustness of this conclusion. Therefore, at the current price, this combination regimen is not cost-effective.

In recent years, multiple real-world studies have further confirmed the efficacy and safety of irinotecan hydrochloride liposome-based regimens in clinical practice, consistent with the findings of pivotal clinical trials. A multicenter retrospective study from Korea demonstrated that irinotecan hydrochloride liposome combined with 5-FU/LV achieved a median overall survival of 9.4 months and a median progression-free survival of 3.5 months with manageable safety in gemcitabine-pretreated patients with metastatic pancreatic cancer (Yoo et al., 2019). Another single-center analysis from Europe reported similar real-world effectiveness and observed an improvement in overall survival among advanced pancreatic cancer patients following the introduction of this regimen as a second-line therapy (Kieler et al., 2020). These real-world evidence enhance the credibility of the survival benefits extrapolated from clinical trial data in our study.

However, significant survival benefit alone is neither a necessary nor sufficient condition for health insurance reimbursement, its economic value must be rigorously assessed. Our threshold analysis indicates that for the HR070803 regimen to meet the current WTP threshold, its price would need to be drastically reduced by approximately 82.6%, from ¥183.22 per mg to ¥31.9 per mg. Future proactive health insurance price negotiations represent a critical pathway to achieving this goal. Furthermore, exploring patient stratification strategies based on biomarkers (e.g., UGT1A1 genotype) could help precisely identify the patient subpopulation that would benefit the most, thereby optimizing healthcare resource allocation and improving the cost-effectiveness of the treatment strategy.

The results of this study should be interpreted within the context of limited treatment options for advanced PDAC and a scarcity of economic evidence in this area. Although several new drugs have emerged in recent years, few are available for second-line treatment and demonstrate significant survival improvement (Catenacci et al., 2015; O'Reilly et al., 2019), leading to a relative paucity of health economic evaluations in this field. A study from the US payer perspective also found that the liposomal irinotecan combination regimen was not cost-effective compared to the control group, with an ICER of $206,340.69 per QALY, far exceeding the local threshold (Shao et al., 2024). Another economic evaluation of the FOLFIRINOX first-line treatment regimen reached a similar conclusion (Attard et al., 2014). This study is the first, based on Chinese data, to arrive at a congruent finding: despite the significant survival benefit offered by irinotecan hydrochloride liposome (HR070803), the substantial incremental cost results in an ICER far exceeding the domestically accepted WTP threshold. This collectively suggests that high drug cost is the primary barrier preventing innovative PDAC treatment regimens from demonstrating cost-effectiveness, a challenge prevalent globally. This global challenge reflects a common dilemma faced by innovative therapies for refractory cancers: high R&D costs, a relatively limited target patient population, and urgent clinical needs collectively drive high drug pricing. Consequently, enhancing the economic value of drugs through strategic purchasing (e.g., insurance negotiations), value-based pricing, and identifying precise beneficiary subpopulations has become a key issue requiring coordinated efforts from healthcare systems and payers worldwide.

In this context, our threshold analysis points to potential pathways for improving the regimen’s economic value. The analysis shows that reducing the price of irinotecan hydrochloride liposome by approximately 82.6% would be necessary to bring the ICER below the WTP threshold of three times per capita GDP (¥268,074 per QALY). This finding aligns closely with the core logic of health insurance negotiations, which aim to lower drug prices through “volume-for-price” agreements, providing a concrete, evidence-based reference point for future negotiations. Additionally, exploring patient stratification based on biomarkers (e.g., UGT1A1 genotype) to accurately identify subgroups deriving the most significant benefit could further optimize resource allocation and improve the cost-effectiveness ratio from the perspective of “enhancing outcomes,” which should be a focus of future research.

This study has several limitations. First, the model relies on clinical trial data rather than real-world data, introducing uncertainty in long-term survival extrapolation. Although we have supplemented our analysis with real-world evidence to support efficacy, the model itself still relies on the extrapolation of survival curves from the PAN-HEROIC-1 trial. Second, the health utility values used in the model were sourced from studies involving non-Chinese populations (Attard et al., 2014); sensitivity analysis indicated that the utility values for progression-free survival (PFS) and progressive disease (PD) states significantly influenced the results, suggesting this assumption might introduce bias. Third, the model assumed all patients received best supportive care (BSC) after progression. However, data from the PAN-HEROIC-1 trial revealed a notably higher proportion of patients in the control arm actually received post-progression antitumor therapy compared to those in the investigational arm (68.5% vs. 51.7%) (Cui et al., 2024b). Considering the reality of clinical practice in advanced PDAC, where a subset of patients (literature reports at least approximately 15%) may still receive third-line chemotherapy after failure of second-line treatment, the BSC assumption in our model may have systematically underestimated the actual medical costs in the control arm. Consequently, this could lead to an overestimation of the incremental cost-effectiveness ratio (ICER), representing an important limitation of our model. Finally, not incorporating all-grade adverse events and their impact on utility values might also have affected the results.

In summary, this study demonstrates that although the irinotecan hydrochloride liposome (HR070803) combination regimen provides clinically meaningful survival improvement for patients with advanced PDAC who have failed first-line chemotherapy, it is not cost-effective from the perspective of the Chinese healthcare system at its current market price. Future real-world studies will help further validate these findings and provide more comprehensive evidence to support healthcare policy decision-making.

## Data Availability

The original contributions presented in the study are included in the article/Supplementary Material, further inquiries can be directed to the corresponding author.
