# Supplementary material for: Cost-effectiveness analysis of irinotecan hydrochloride liposome in combination with 5-fluorouracil and leucovorin in locally advanced or metastatic pancreatic ductal adenocarcinoma
Source: Front Pharmacol. 2025 Nov 12;16:1596658. doi: 10.3389/fphar.2025.1596658 (PMC12646928; doi:10.3389/fphar.2025.1596658)
Supplement: Supplementary file 3 [file Supplementaryfile1.docx]

install.packages("survival")

install.packages("survminer")

install.packages("survHE")

library(survival)

library(survminer)

library(survHE)

surv_inp<-"T.txt"

nrisk_inp<-"IPD T.txt"

km_out<-"KMapat.txt"

ipd_out<-"IPDapat.txt"

digitise(surv_inp,nrisk_inp,km_output="KMapat.txt",ipd_output="IPDapat.txt")

surv_inp<-"P.txt"

nrisk_inp<-"IPD P.txt"

km_out<-"KMplace.txt"

ipd_out<-"IPDplace.txt"

digitise(surv_inp,nrisk_inp,km_output="KMplace.txt",

ipd_output="IPDplace.txt")

ipd_filesa<-list("IPDapat.txt")

ipd_filesp<-list("IPDplace.txt")

ipd_files<-list("IPDplace.txt","IPDapat.txt")

apat<-make.ipd(ipd_filesa,var.labs = c("time","event","arm"))

place<-make.ipd(ipd_filesp,var.labs = c("time","event","arm"))

data<-make.ipd(ipd_files,ctr=1,var.labs = c("time","event","arm"))

fit<-survfit(Surv(time,event==1)~arm,data =data)

fit

ggsurvplot(fit,data=data,

pval =F,

risk.table = TRUE,

legend.title="Interventions",

legend.labs=c("S1","Irinotecan+S1"),

risk.table.height=0.2,

xlab=c("Time since randomization (months)"),

ylab=c("progression-free survival(probability"),

risk.table.col="strata",

tables.theme=theme_cleantable(),

ggtheme=theme_classic())

mods1<-c("exponential","gompertz","weibull","weibullPH","loglogistic","lognormal")

formula<-Surv(time,event)~1

apatinib_mle<-fit.models(formula=formula,data=apat,distr=mods1,method="mle")

apatinib_mle

place_mle<-fit.models(formula=formula,data=place,distr=mods1,method="mle")

print(apatinib_mle,mod=5)

print(place_mle,mod=6)

apatinib_mle$models

place_mle$models

plot(apatinib_mle,cex.trt=0.5)

plot(apatinib_mle,xlab="time(mouths",ylab="overall survival",add.km=T)

plot(place_mle,cex.trt=0.5)

plot(place_mle,xlab="time(mouths",ylab="overall survival(%)",add.km=T)

apatinib_mle$models.fitting

place_mle$models.fitting

model.fit.plot(apatinib_mle,type = "aic",scale="relative")

model.fit.plot(place_mle,type = "aic",scale="relative")

psa=make.surv(apatinib_mle,mod=3,nsim=1000)
